# Islet-1 promotes the cardiac-specific differentiation of mesenchymal stem cells through the regulation of histone acetylation

**DOI:** 10.3892/ijmm.2014.1687

**Published:** 2014-03-06

**Authors:** NAIJING YIN, RONG LU, JIANPING LIN, SHENSHEN ZHI, JIE TIAN, JING ZHU

**Affiliations:** 1 Ministry of Education Key Laboratory of Child Development and Disorders, Pediatric Research Institute, Children’s Hospital of Chongqing Medical University, Chongqing 400014, P.R. China; 2 Cardiovascular Department (Internal Medicine), Children’s Hospital of Chongqing Medical University, Chongqing 400014, P.R. China

**Keywords:** mesenchymal stem cells, cardiomyocyte, differentiation, histone acetylation

## Abstract

The aim of the present study was to investigate the effects of Islet-1 on the process of mesenchymal stem cell (MSC) differentiation into cardiomyocyte-like cells and to elucidate the possible mechanisms involved. Lentiviral vectors expressing Islet-1 (Lenti-Islet-1) were constructed and used for C3H10T1/2 cell transfection. Cell morphology was observed. Cardiac-related genes and proteins were detected by qPCR and western blot analysis. Epigallocatechin gallate (EGCG) was used as an inhibitor of acetylated histone H3 (AcH3). AcH3 was detected by chromatin immunoprecipitation. Cells overexpressing Islet-1 tended to change into fibroblast-like cells and were arranged in the same direction. The enhanced expression of GATA binding protein 4 (Gata4), NK2 homeobox 5 (Nkx2.5), myocyte enhancer factor 2C (Mef2c) and cardiac troponin T (cTnT) was observed in the cells overexpressing Islet-1 following transfection with Lenti-Islet-1. However, the expression of hepatocyte-, bone- and neuronal-specific markers was not affected by Islet-1. The AcH3 relative amount increased following transfection with Lenti-Islet-1, which was associated with the enhanced expression of Gata4, Nkx2.5 and Mef2c in these cells. The expression of Gata4, Nkx2.5 and Mef2c in the C3H10T1/2 cells transfected with Lenti-Islet-1 and treated with EGCG was reduced following treatment with EGCG. The data presented in this study indicate that Islet-1 specifically induces the differentiation of C3H10T1/2 cells into cardiomyocyte-like cells, and one of the mechanisms involved is the regulation of histone acetylation.

## Introduction

Heart failure caused by cardiac cell death is a growing health issue worldwide. Despite various treatment options, the rising prevalence of this disease renders it one of the leading causes of morbidity and mortality. Stem cell transplantation, a promising treatment for heart failure, has been successfully applied on experimental animals to enhance myocardial regeneration and improve cardiac function (1).

Over the years, a number of experimental trails have focused on mesenchymal stem cells (MSCs) as they are easily available and have the capacity to differentiate into cardiomyocyte-like cells (2–4). Studies have indicated that the regulation of histone acetylation and deacetylation plays an important role in the process of MSC transformation into cardiomyocyte-like cells (5–7). Histone acetylation or deacetylation enhances the expression of cardiac-specific genes (8,9).

The exact mechanisms responsible for histone acetylation and deacetylation during the differentiation of MSCs into cardiomyocyte-like cells has not been determined yet. A number of studies have reported that the histone acetylases (HATs) and deacetylases (HADCs) lack the cardiac-specific gene binding sites (10,11). Therefore, HATs and HADCs cannot specifically bind to cardiac-specific genes and regulate their expression directly. However, Islet-1, a subtype of the LIM-homeodomain transcription factor (LIM-HD) subfamily, contains a DNA binding site and two LIM domains, and is able to bind with GCN5, which is a member of the HAT family involved in cardiomyocyte, hepatocyte, as well as bone and neuron differentiation (12–14). Several studies have demonstrated that Islet-1 is crucial to cardiac development and cardiomyocyte differentiation (15–16). Thus, we hypothesized that Islet-1 is a key factor during the process of the specific differentiation of MSCs into cardiomyocyte-like cells and that it exerts its effects possibly through modifications in histone acetylation and deacetylation. In this study, in order to verify our hypothesis we used C3H10T1/2 cells overexpressing Islet-1 to determine cell differentiation and the expression of heart development-related genes in these cells. Our data indicate that Islet-1 enhances cardiac cell differentiation and increases the expression of heart development-related genes, possibly through the regulation of histone acetylation.

## Materials and methods

### Construction of lentiviral vectors carrying the Islet-1 gene (Lenti-Islet-1)

Mouse Islet-1 cDNA (NM_021459) was obtained through the reverse transcription polymerase chain reaction (RT-PCR) (RT-PCR system; Bio-Rad, Hercules, CA, USA) of total RNA isolated from 13.5-day-old mouse hearts using the RNA extraction kit (RP120; BioTeke, Beijing, China), SYBR Premix Ex Taq II and PrimeScript RT reagent kit (Takara, Dalian, China). The Islet-1 gene primer (5′-cggatcctacagatatggg agacatgggcgatc-3′ and 5′-cgtcgactcctcatgccctcaataggactgg-3′), was designed by Primer Premier 5.0 software (Premier Biosoft, Palo Alto, CA, USA) and synthesized by Sangon Biotech Co., Ltd. (Shanghai, China). RT-PCR was performed as follows: 95ºC (10 min), 50ºC (30 sec) and 72ºC (1 min), 35 cycles.

The Islet-1 gene and pWPI-green fluorescent protein (GFP) plasmid (Invitrogen, Grand Island, NY, USA) were double digested with restriction endonuclease (Takara). The product was purified, recombined directly and transformed into *E. coli*-competent cells (DH5α; Invitrogen). The positive clone with the Islet-1 gene was connected to the right vector and identified by PCR. The pWPI-GFP-Islet-1 plasmid was sequenced and compared with the mouse Islet-1 gene.

The packaging and production of the lentiviral vector were carried out as previously described (17). Briefly, the lentiviral shuttle plasmid and auxiliary packaging plasmid were constructed. The 293T cells [Institute of Biochemistry and Cell Biology (SIBS) of the Chinese Academy of Sciences (CAS, Shanghai, China)] were co-transfected with the lentivirus shuttle plasmid and auxiliary packaging plasmid. The culture medium was replaced by Dulbecco’s modified Eagle’s medium (DMEM; Thermo Fisher Scientific, Waltham, MA, USA) 8 h following transfection. Lentiviral vectors were collected from the supernatant 48 h after the medium exchange.

### Cell culture and lentiviral vector transfection

The C3H10T1/2 cells (University of Chicago Molecular Oncology Laboratory, Chicago, IL, USA) were grown in DMEM supplemented with 10% fetal bovine serum (FBS; HyClone, Philadelphia, PA, USA).

Lentiviral vectors [vectors with pWPI-GFP-Islet-1 plasmid (Lenti-Islet-1) or vectors with pWPI-GFP plasmid alone (Lenti-N), respectively, MOI=20] and polybrene were added to the C3H10T1/2 cells dissociated with trypsinase (Invitrogen), while the density of the cells was 60%, with a concentration of 8 mg/l. The culture medium was replaced by DMEM with 10% FBS after 12 h of incubation at 37ºC, 5% CO_2_. Fluorescence microscopy (BX51; Olympus, Tokyo, Japan) was used to observe GFP expression after 3 days. Flow cytometry (FCM) (BD Canto II Flow Cytometer; BD Biosciences, San Jose, CA, USA) was used to detect the transfection efficiency.

### RNA isolation, RT-PCR and qPCR

RNA from the untreated C3H10T1/2 cells, the C3H10T1/2 cells transfected with Lenti-N and the C3H10T1/2 cells transfected with Lenti-Islet-1 was isolated using the RNA extraction kit (RP120; BioTeke). The concentration of RNA was determined using a NanoDrop 1000 spectrophotometer (NanoDrop 1000; NanoDrop Products, Wilmington, DE, USA). cDNA was generated using the PrimeScript RT reagent kit (Takara) for RT-PCR (Invitrogen). RT-PCR was performed at 30ºC (10 min), 42ºC (30 min), 99ºC (5 min) and 5ºC (5 min). cDNA was analyzed by qPCR (CFX 96 Real-Time System; Bio-Rad, Hercules, CA, USA) using SYBR-Green RealMasterMix (Tiangen Biotech, Co., Inc., Beijing, China). The primer sequences, product size and annealing temperatures are presented in Table I.

qPCR was performed, including an initial denaturation at 98ºC (5 min), followed by denaturation at 94ºC (10 sec) and annealing at the annealing temperature indicated in Table I (10 sec). This was finally followed by a renaturation at 72ºC (15 sec) and then the process was repeated for 40 cycles.

### Western blot analysis and immunofluorescence

Proteins (20 μg) were loaded onto 8% SDS-polyacrylamide gels for electrophoresis and then the proteins were then transferred onto nitrocellulose membranes. The transferred nitrocellulose membranes were blocked with 5% dried skim milk for 1 h at room temperature, then incubated with primary antibodies at 4ºC for 12 h, then incubated with the secondary antibodies in blocking buffer for 1 h. The bands were revealed by enhanced chemiluminescence reagents (Santa Cruz Biotechnology, Inc., Dallas, TX, USA) for 1 min and analyzed using Image-Pro Plus 5.1 software (Media Cybernetics, Inc., Rockville, MD, USA). The antibodies used were as follows: anti-Islet-1 (sc-23590; Santa Cruz Biotechnology, Inc.), mouse monoclonal to cardiac troponin T (cTnT; ab33589), albumin (ALB), bone-specific alkaline phosphatase (BALP), glial fibrillary acidic protein (GFAP) and rabbit polyclonal to histone H3-ChIP Grade (ab1791) (all from Abcam, Cambridge, UK).

Cells (4.4×10^5^/well) were plated in 6-well plates on 1×1 cm^2^ glass coverslips. They were later fixed in 4ºC acetone in 15 min. Following 3 washes in PBS, the cells on glass coverslips were blocked with goat serum (1:20), washed again, then primary Islet-1 monoclonal antibody (1:100; Santa Cruz Biotechnology, Inc.) was added for overnight incubation at 4ºC. The cells were then washed with PBS and secondary antibodies (1:150; CoWin Bioscience, Beijing, China) conjugated with TRITC were added followed by incubation for 1 h at 37ºC. DAPI was then added for 3 min. After the final wash, images were acdquired under a fluorescence microscope (BX51; Olympus).

### Epigallocatechin gallate (EGCG) treatment

EGCG (120 μmol/l) (Sigma-Aldrich, St. Louis, USA) was added to the C3H10T1/2 cells transfacted with Lenti-Islet-1 2 weeks following transfection. Total RNA from these cells was isolated 3, 6 and 12 h following treatment with EGCG. RT-PCR and qPCR were performed to detect GATA binding protein 4 (Gata4), NK2 homeobox 5 (Nkx2.5) and myocyte enhancer factor 2C (Mef2c) expression.

### Chromatin immunoprecipitation (ChIP)-qPCR

Chromatin samples were prepared from the cells in the C3H10 group (untransfected cells), the negative control group (cells transfected with Lenti-N) and the experimental group (cells transfected with Lenti-Islet-1). ChIP assay was performed as previously described in the study by Zsindely *et al* (18). Briefly, chromatin samples were cross-linked with 1% formaldehyde then fragmented by sonication (Ultrasonic Disruptor UD-201; CS Bio Co., Menlo Park, CA, USA). Agarose gel electrophoresis was carried out to verify the length of the DNA fragments. Immunoprecipitation was performed using rabbit polyclonal to histone H3-ChIP Grade antibody (ab1791; Abcam). The chromatin-antibody complexes were then washed, reverse cross-linked and purified. The ChIP process was performed using the Chromatin Immunoprecipitation kit (Millipore, Billerica, MA, USA). The amount of extracted DNA was determined by qPCR. ChIP-qPCR primers were designed using Primer Premier 5.0 software and synthesized by Shanghai DNA Biotechnologies Co., Ltd. The primer sequences, product size and annealing temperatures of the ChIP-qPCR reaction are presented in Table II.

### Statistical analysis

All the data are expressed as the means ± standard error of the mean (SEM) and were analyzed with repeated measures ANOVA (TCDD data). A test for linear trend and Dunnett’s test (comparison of all treated groups with controls) were used as post-tests in ANOVA. SPSS 17.0 software (SPSS Inc., Armonk, NY, USA) was used for statistical analyses. A value of P<0.05 was considered to indicate a statistically significant difference.

## Results

### Construction of lentiviral vectors

**Figure 1:**
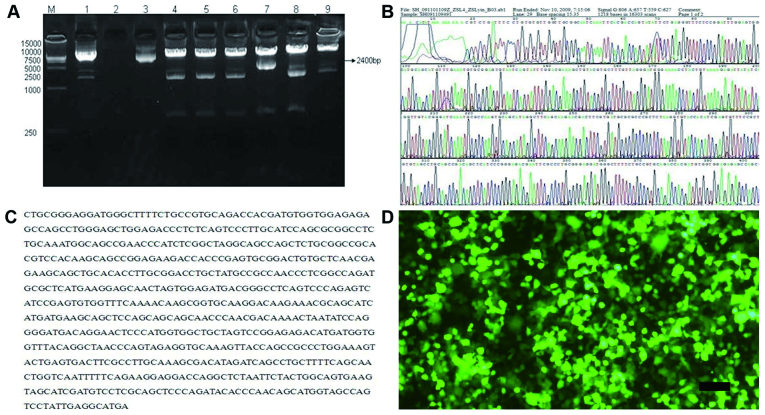
(A) Detection of lentiviral vector. PCR of random clones. Lane M, DL15000 DNA marker; lanes 1–9, clones that we selected. The 7th lane shows the positive clone. (B and C) Part of the Lenti-Islet-1 plasmid sequencing result. (D) Detection of green fluorescent protein (GFP) expression in 293T cells following transfection with Lenti-Islet-1 vectors (magnification, ×10). Scale bar, 100 μm.

Following double digestion with the pWPI vector, the negative fragment was in the vicinity of 1,400 bp and the positive fragment was 2,400 bp. Fragment 7 (2,400 bp) was the positive clone, identified by PCR (Fig. 1A). Sequencing analysis displayed the positive clone insertion into the pWPI vector (Fig. 1B and C). On the 4th day after Lenti-Islet-1 transfection, GFP expression could be detected in the 293T cells (Fig. 1D).

### Transfection efficiency and Islet-1 expression

**Figure 2:**
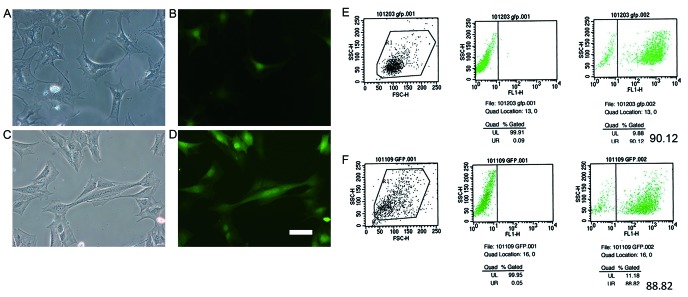
Green fluorescent protein (GFP) expression detected under a fluorescence microscope. The lentiviral vectors carried the GFP gene; thus, a fluorescence microscope was used to detected GFP expression in the C3H10T1/2 cells transfected with Lenti-N and the C3H10T1/2 cells transfected with Lenti-Islet-1 at 3 days after transfection. (A) Cell morphology of the C3H10T1/2 cells transfected with Lenti-N observed under a microscope (magnification, ×20). (B) GFP expression of the C3H10T1/2 cells transfected with Lenti-N observed udner a fluorescence microscope (magnification, ×20). (C) Cell morphology of the C3H10T1/2 cells transfected with Lenti-Islet-1 observed under a microscope (magnification, ×20). (D) Cell morphology of the C3H10T1/2 cells transfected with Lenti-Islet-1 observed under a microscope (magnification, ×20). Scale bar, 20 μm. (E and F) Transfection efficiency of teh C3H10T1/2 cells transfected with Lenti-N and the C3H10T1/2 cells transfected with Lenti-Islet-1 detected by flow cytometry (FCM). The transfection efficiency of the C3H10T1/2 cells transduced with lentiviral vectors with pWPI-GFP plasmid or lentiviral vectors with pWPI-GFP-Islet-1 plasmid was detected by FCM. (E) The transfection efficiency of the C3H10T1/2 cells transfected with Lenti-N was 90.12%. (F) The transfection efficiency of the C3H10T1/2 cells transfected with Lenti-Islet-1 was 88.82%.

Since the vectors carried the pWPI-GFP plasmid, GFP could be observed under a fluorescence microscope in both the Lenti-Islet-1- and Lenti-N-transfected cells. Our data indicated that GFP could be observed in the C3H10T1/2 cells transfected with Lenti-N (Fig. 2A and B) and Lenti-Islet-1 (Fig. 2C and D) 3 days after transfection. The transfection efficiencies of the C3H10T1/2 cells transfected with Lenti-N and Lenti-Islet-1 were determined by FCM. The transfection efficiency of the C3H10T1/2 cells transfected with Lenti-N was 90.12% (Fig. 2E) and that of the C3H10T1/2 cells transfected with Lenti-Islet-1 was 88.82% (Fig. 2F).

**Figure 3:**
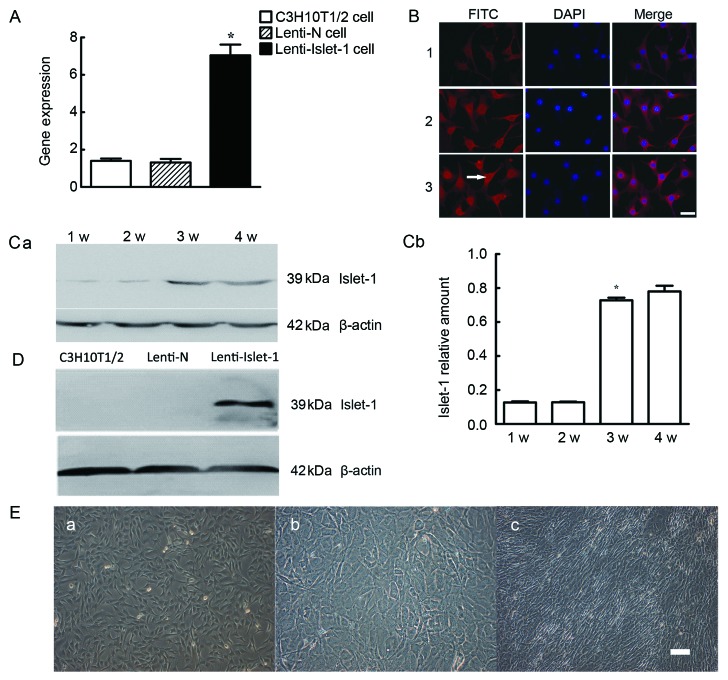
(A) Islet-1 gene expression in the C3H10T1/2 cells transfected with Lenti-Islet-1 was higher (^*^P<0.05) than that in the other C3H10T1/2 cells. (B) The expression of Islet-1 was detected by immunofluorescence in each group: Panel 1, untransfected C3H10T1/2 cells; panel 2, C3H10T1/2 cells transfected with Lenti-N; panel 3, C3H10T1/2 cells transfected with Lenti-Islet-1. FITC staining of Islet-1 protein showing its location. DAPI staining of cell nucleus. Merge image was obtained by overlapping FITC and DAPI images (magnification, ×20). Scale bar, 20 μm. (C-a) The expression of Islet-1 at different time points in the C3H10T1/2 cells transfected with Lenti-Islet-1: 1w, 1st week; 2w, 2nd week; 3w, 3rd week; 4w, 4th week. (C-b) Islet-1 protein expression in the C3H10T1/2 cells transfected with Lenti-Islet-1 was higher during the 3rd week than in the first 2 weeks (^*^P<0.05). (D) Islet-1 protein expression in the C3H10T1/2 cells transfected with Lenti-Islet-1 was higher than that in the untransfected C3H10T1/2 cells and the C3H10T1/2 cells transfected with Lenti-N. (E) Morphological changes of C3H10T1/2 cells transfected with lentiviral vectors with pWPI-GFP plasmid or lentiviral vectors with pWPI-GFP-Islet-1 plasmid observed under a microscope. No difference was observed between the untransfected C3H10T1/2 cells (magnification, ×10) (E-a) and the C3H10T1/2 cells transfected with Lenti-N (magnification, ×10) (E-b), whereas the C3H10T1/2 cells transfected with Lenti-Islet-1 (×10) (E-c) turned into fibroblast-like cells and were arranged toward the same direction. Scale bar, 100 μm.

The results from PCR revealed that the expression of the Islet-1 gene in the Lenti-Islet-1-transfected cells was higher than that in the untransfected cells (C3H10T1/2 cells) and those transfected with Lenti-N (P<0.05) (Fig. 3A). Islet-1 protein was expressed in the cytoplasm. The fluorescence intensity in the C3H10T1/2 cells transfected with Lenti-Islet-1 was higher than that in the untransfected cells (C3H10T1/2 cells) and the C3H10T1/2 cells transfected with Lenti-N (Fig. 3B). The expression of Islet-1 protein in the Lenti-Islet-1-transfected cells increased progressively with the highest expression observed at 3 and 4 weeks after transfection (Fig. 3C). Western blot analysis revealed that the expression of Islet-1 protein in the Lenti-Islet-1-transfected cells was higher than that in the untransfected C3H10T1/2 cells and those transfected with Lenti-N (Fig. 3D).

### Islet-1 promotes the specific differentiation of C3H10T1/2 cells into cardiomyocyte-like cells

Under microscopic observation, no difference in cell morphology was observed between the C3H10T1/2 cells not transfected with lentivirus (Fig. 3E-a) and those transfected with Lenti-N (Fig. 3E-b). However, the C3H10T1/2 cells transfected with Lenti-Islet-1 (Fig. 3E-c) turned into fibroblast-like cells and were arranged in the same direction.

**Figure 4:**
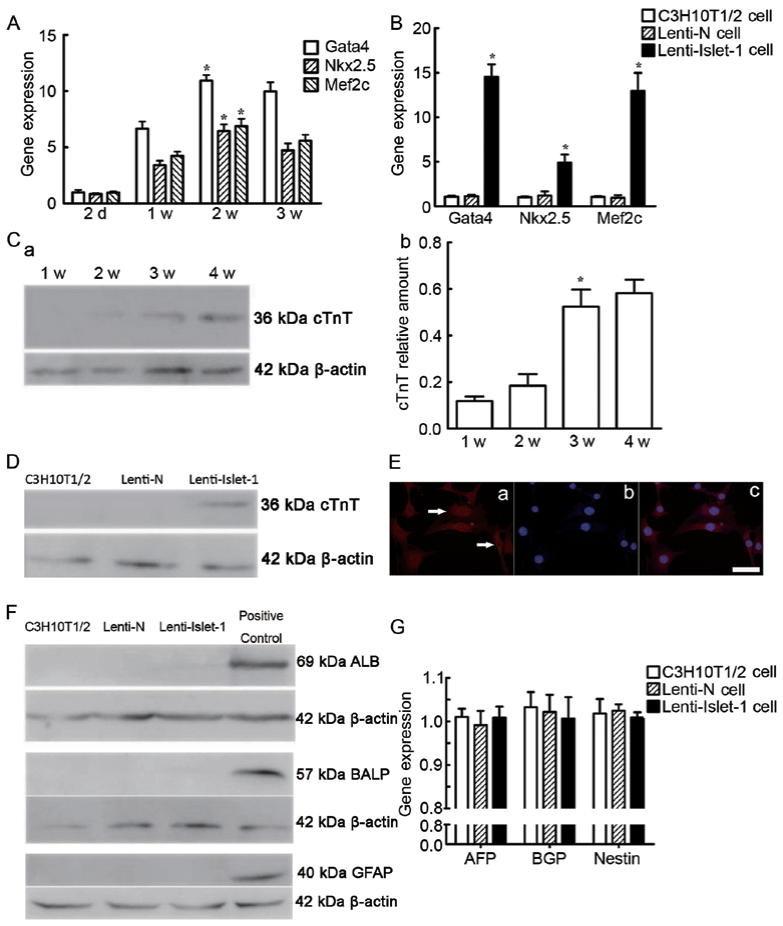
(A) The expression of Gata4, Nkx2.5 and Mef2c gene at different time points in C3H10T1/2 cells transfected with Lenti-Islet-1. 2d, 2nd day; 1w, 1st week; 2w, 2nd week; 3w, 3rd week. The peak expression of cardiac-specific genes in the C3H10T1/2 cells transfected with Lenti-Islet-1 was significantly higher than that in the untransfected C3H10T1/2 cells and the C3H10T1/2 cells transfected with Lenti-N at 2 weeks after transfection (^*^P<0.05). (B) The expression of cardiac-specific genes in the C3H10T1/2 cells transfected with Lenti-Islet-1 was higher in the 2nd week than the other time points (^*^P<0.05). (C) The protein expression of cTnT at different time points in the C3H10T1/2 cells transfected with Lenti-Islet-1. 1w, 1st week; 2w, 2nd week; 3w, 3rd week; 4w, 4th week. cTnT expression increased from the 3rd week following transfection (^*^P<0.05). (D) cTnT expression in the untransfected C3H10T1/2 cells, the C3H10T1/2 cells transfected with Lenti-N and the C3H10T1/2 cells transfected with Lenti-Islet-1 at 3 weeks after transfection. cTnT expression was higher in the C3H10T1/2 cells transfected with Lenti-Islet-1 than the other 2 groups of cells. (E) cTnT expression in the C3H10T1/2 cells transfected with Lenti-Islet-1 was located in the cell nucleus and cytoplasm (magnification, ×20). Scale bar, 20 μm. (F) Expression of hepatocyte-, bone- and neuronal-specific markers. Albumin (ALB), bone-specific alkaline phosphatase (BALP) and glial fibrillary acidic protein (GFAP) expression was detected by western blot analysis in the untransfected C3H10T1/2 cells, the C3H10T1/2 cells transfected with Lenti-N and the C3H10T1/2 cells transfected with Lenti-Islet-1 and the positive control group. There was no ALB, BALP and GFAP expression observed in the untransfected C3H10T1/2 cells, the C3H10T1/2 cells transfected with Lenti-N and the C3H10T1/2 cells transfected with Lenti-Islet-1. (G) AFP, bone Gla protein (BGP) and nestin expression detected by qPCR. The expression of BGP, AFP and nestin was did not differ significantly between the 3 groups.

Cardiac-specific genes, such as Gata4, Nkx2.5 and Mef2c, were detected by qPCR following transfection with the lentiviral vectors. The expression of these genes in the C3H10T1/2 cells transfected with Lenti-Islet-1 was higher during the 2nd week following transfection than the 1st week and 3rd week (Fig. 4A). The peak expression of the cardiac-specific genes in the C3H10T1/2 cells transfected with Lenti-Islet-1 was markedly higher than that in the untransfected C3H10T1/2 cells and those transfected with Lenti-N 2 weeks following transfection (Fig. 4B). The expression of cTnT increased from the 3rd week following transfection and not during the first 2 weeks (Fig. 4C). The expression of cTnT was higher in the C3H10T1/2 cells transfected with Lenti-Islet-1 than in the other 2 groups at 3 weeks following transfection (Fig. 4D). cTnT in the C3H10T1/2 cells transfected with Lenti-Islet-1 was located in the cell nucleus and cytoplasm (Fig. 4E).

The hepatocyte-specific markers, α-fetoprotein (AFP) and ALB; the bone-specific markers, bone Gla protein (BGP) and BALP; the neuronal specific markers, nestin and GFAP, were detected by western blot analysis or qPCR. There was no ALB, BALP or GFAP expression observed in the C3H10T1/2 cells, the C3H10T1/2 cells transfected with Lenti-N and the C3H10T1/2 cells transfected with Lenti-Islet-1 (Fig. 4F). The expression of BGP, AFP and nestin did not differ between the 3 groups of cells (Fig. 4G).

### Changes in histone acetylation status induced by Islet-1 overexpression

**Figure 5:**
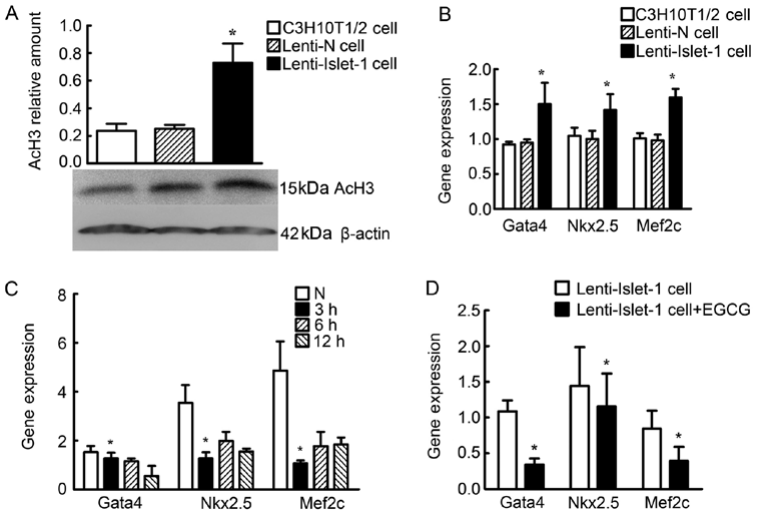
Differences in histone acetylation levels in the untransfected C3H10T1/2 cells, the C3H10T1/2 cells transfected with Lenti-N and the C3H10T1/2 cells transfected with Lenti-Islet-1. (A) Acetylated histone H3 (AcH3) was detected by western blot analysis in the untranstected C3H10T1/2 cells, the C3H10T1/2 cells transfected with Lenti-N and the C3H10T1/2 cells transfected with Lenti-Islet-1. The AcH3 relative amount in the C3H10T1/2 cells transfected with Lenti-Islet-1 was higher than that in the untransfected C3H10T1/2 cells and the C3H10T1/2 cells transfected with Lenti-N (^*^P<0.05). (B) Chromatin immunoprecipitation (ChIP) and qPCR were performed to reveal the acetylation levels of histone H3 on the cardiac-specific genes, GATA binding protein 4 (Gata4), NK2 homeobox 5 (Nkx2.5) and myocyte enhancer factor 2C (Mef2c), at their peak expression times (2 weeks after transfection). The expression of Gata4, Nkx2.5 and Mef2c combined with AcH3 in the C3H10T1/2 cells transfected with Lenti-Islet-1 was higher than that in the untransfected C3H10T1/2 cells and the C3H10T1/2 cells transfected with Lenti-N (^*^P<0.05). (C) Gata4, Nkx2.5 and Mef2c expression was found to be reduced 3 h following treatment with 120 μmol/l epigallocatechin gallate (EGCG) (^*^P<0.05). (D) Gata4, Nkx2.5 and Mef2c expression in the C3H10T1/2 cells transfected with Lenti-Islet-1 and treated with EGCG was lower compared with the cells not treated with EGCG (^*^P<0.05).

Acetylated histone H3 (AcH3) was detected by western blot analysis in the untransfected C3H10T1/2 cells (controls), the C3H10T1/2 cells transfected with Lenti-N and the C3H10T1/2 cells transfected with Lenti-Islet-1. The relative amount of AcH3 in the C3H10T1/2 cells transfected with Lenti-Islet-1 was higher than that of the other cells (Fig. 5A). ChIP and qPCR were performed to determine the acetylation levels of histone H3 at the cardiac-specific genes, Gata4, Nkx2.5 and Mef2c, at their peak expression times (2 weeks after transfection). The expression of Gata4, Nkx2.5 and Mef2c combined with AcH3 in the C3H10T1/2 cells transfected with Lenti-Islet-1 was higher than that in the untransfected C3H10T1/2 cells and in the C3H10T1/2 cells transfected with Lenti-N (Fig. 5B). Gata4, Nkx2.5 and Mef2c expression was found to be reduced 3 h following treatment with 120 μmol/l EGCG (Fig. 5C). Gata4, Nkx2.5 and Mef2c expression in the C3H10T1/2 cells transfected with Lenti-Islet-1 and treated with EGCG was lower compared with the cells not treated with EGCG (Fig. 5D).

## Discussion

Stem cells have multiple differentiation potencies and immunological features that render them promising candidates in cell transplantation as a therapeutic option. Stem cells can specifically differentiate into various cell types under different treatment conditions. For example, MSCs treated with 5-azacytidine can differentiate into cardiomyocyte-like cells (19). Endothelial progenitor cells (EPCs) induced by vascular endothelial growth factor A (VEGF-A) can differentiate into vascular endothelial cells (20). Stem cell-specific differentiation is a complex process, and the mechanisms involved are still unknown.

Histone acetylation and deacetylation are critical to the modification of chromatin structure associated with the regulation of gene expression (21). Acetylation and deacetylation is involved in various developmental processes, including heart development. A number of studies have revealed that histone acetylation and deacetylation play an important role in the differentiation of stem cells (22). The expression of cardiac-related genes, such as Gata4, Nkx2.5, Mef2cj and cTnT is reduced following interference of GCN5, a key histone acetyltransferase (23). Previous studies have demonstrated that the expression of cardiac-related genes (both at the transcriptional and translational level) is increased in C3H10T1/2 cells following treatment with trichostatin A [TSA, a histone deacetylase (HADC) inhibitor] (24,25).

However, neither GCN5 nor other HATs/HADCs posses DNA binding sites. The regulatory mechanism through histone acetylation or deacetylation seems not to be specific. GCN5 generally binds to the specific target DNA sequence requiring other factors that have DNA-banding domains (26).

To reveal the factors that are associated with HATs and HADCs, studies have screened and analyzed the GCN5 protein complexes in the process when MSCs specifically differentiate into cardiomyocyte-like cells. LIM-HD, fibroblast growth factor-14 (FGF-14), leucine zipper protein 1 (LUZP1), cyclin-L1, NF-κB inhibitor α and Kruppel-like factor 10 have been suggested as the co-factors of GCN5, indicating that GCN5 is involved in cardiomyocyte, hepatocyte, bone and neuronal differentiation (27,28).

Islet-1, which contains one DNA binding site and two LIM domains is a subtype of the LIM-HD subfamily, which is critical for heart development. Islet-1 is located in the second heart field and the outflow tract in the embryonic heart. Various types of congenital heart disease can be caused by Islet-1 insufficiency (14,15,29). Islet-1 has also been recognized as a marker of cardiovascular progenitors (16,30). Islet-1-positive progenitors can differentiate into diverse cardiovascular cell lineages.

In the present study, Islet-1 expression vectors were generated and then successfully transfected into C3H10T1/2 cells. Our data indicate that Islet-1 is a key factor in acetylation during the process of heart development and the MSC-specific differentiation into cardiomyocyte-like cells. Islet-1 specifically promotes the differentiation of C3H10T1/2 cells into cardiomyocyte-like cells through histone acetylation. C3H10T1/2 cells can be specifically induced to differentiate into cardiomyocyte-like cells by Islet-1 overexpression. However, the expression of hepatocyte-, bone- and neuronal-specific markers is not affected by Islet-1. At least one of the mechanisms responsible for the Islet-1-induced differentiation of C3H10T1/2 into cardiomyocyte-like cells is the regulation of histone acetylation. Islet-1, an important co-factor in histone acetylation, may promote the cardiac-specific differentiation of stem cells. The results obtained from the present study provide useful information regarding the clinical application of stem cells in cell transplantation therapy and may offer unique opportunities for the prevention and treatment of heart diseases caused by cardiac cell death.

## Figures and Tables

**Table I:** Primer sequences, product size and annealing temperatures used in qPCR.

Gene	Primer sequence	Product size (bp)	Annealing temperature (ºC)
Islet-1	5′-acaccttgggcggacctgctatg-3′ 5′-tgaaaccacactcggatgactctg-3′	123	60
Gata4	5′-ctgtggcctctaccacaagatgaa-3′ 5′-gtctggcagttggcacagga-3′	180	60
Nkx2.5	5′-acttgtctcctcggtgcttctg-3′ 5′-cacagggttagggtgggactatg-3′	173	63.7
Mef2c	5′-gcgaaagttcggattgatgaaga-3′ 5′-gtggatgtcagtgctggcgta-3′	133	60
AFP	5′-ggaagatggtgagcattg-3′ 5′-tgttggaatacgaagagttg-3′	168	60
BGP	5′-aacgcatctacggtatca-3′ 5′-gctgtgacatccatacttg-3′	134	63
Nestin	5′-atgagcagatgacagtga-3′ 5′-tccagtgattctatgttctct-3′	181	60
β-actin	5′-ggagattactgccctggctccta-3′ 5′-gactcatcgtactcctgcttgctg-3′	50	60

Gata4, GATA binding protein 4; Nkx2.5, NK2 homeobox 5; Mef2c, myocyte enhancer factor 2C; AFP, α-fetoprotein; BGP, bone Gla protein.

**Table II:** Primer sequences, product size and annealing temperatures used in ChIP-qPCR.

Gene	Primer sequences	Product size (bp)	Annealing temperature (ºC)
Gata4	5′-cactgacgccgactccaaactaa-3′ 5′-cgactggggtccaatcaaaag-3′	140	60
Nkx2.5	5′-cttctggctttcaatccatcctca-3′ 5′-cgggcagttctgcgtcaccta-3′	289	60
Mef2c	5′-cacgcatctcaccgcttgacg-3′ 5′-caccagtgcctttctgcttctcc-3′	217	68

Gata4, GATA binding protein 4; Nkx2.5, NK2 homeobox 5; Mef2c, myocyte enhancer factor 2C.

## References

[1] Christoforou N, Gearhart JD (2007). Stem cells and their potential in cell-based cardiac therapies. Prog Cardiovasc Dis.

[2] Bianco P, Robey PG, Simmons PJ (2008). Mesenchymal stem cells: revisiting history, concepts, and assays. Cell Stem Cell.

[3] Pittenger MF, Mackay AM, Beck SC (1999). Multilineage potential of adult human mesenchymal stem cells. Science.

[4] Qin JJ, Xian SX, Huang XW, Sun JH (2011). Differentiation of mesenchymal stem cells into cardiomyocyte-like cells in vitro: Drug, microenvironment and method. Zhongguo Zuzhi Gongcheng Yanjiu Yu Linchuang Kangfu.

[5] Mafi1 P, Hindocha S, Mafi R, Griffin M, Khan WS (2011). Adult mesenchymal stem cells and cell surface characterization - a systematic review of the literature. Open Orthop J.

[6] Psaltis PJ, Zannettino AC, Worthley SG, Gronthos S (2008). Concise review: mesenchymal stromal cells: potential for cardiovascular repair. Stem Cells.

[7] Verdone L, Caserta M, Di Mauro E (2005). Role of histone acetylation in the control of gene expression. Biochemistry and cell biology. Biochem Cell Biol.

[8] Gupta MP, Samant SA, Smith SH, Shroff SG (2008). HDAC4 and PCAF bind to cardiac sarcomeres and play a role in regulating myofilament contractile activity. J Biol Chem.

[9] Yang G, Tian J, Feng C, Zhao LL, Liu Z, Zhu J (2012). Trichostatin a promotes cardiomyocyte differentiation of rat mesenchymal stem cells after 5-azacytidine induction or during coculture with neonatal cardiomyocytes via a mechanism independent of histone deacetylase inhibition. Cell Transplant.

[10] Shahbazian MD, Grunstein M (2007). Functions of site-specific histone acetylation and deacetylation. Annu Rev Biochem.

[11] Wang Z, Zang C, Cui K (2009). Genome-wide mapping of HATs and HDACs reveals distinct functions in active and inactive genes. Cell.

[12] Brade T, Gessert S, Kühl M, Pandur P (2007). The amphibian second heart field: Xenopus islet-1 is required for cardiovascular development. Dev Biol.

[13] Bu L, Jiang X, Martin-Puig S (2009). Human ISL1 heart progenitors generate diverse multipotent cardiovascular cell lineages. Nature.

[14] Yang L, Cai CL, Lin L (2006). Isl1Cre reveals a common Bmp pathway in heart and limb development. Development.

[15] Laugwitz KL, Moretti A, Caron L, Nakano A, Chien KR (2008). Islet1 cardiovascular progenitors: a single source for heart lineages?. Development.

[16] Nakano A, Nakano H, Chien KR (2008). Multipotent islet-1 cardiovascular progenitors in development and disease. Cold Spring Harb Symp Quant Biol.

[17] Toscano MG, Frecha C, Ortega C, Santamaria M, Martin F, Molina IJ (2004). Efficient lentiviral transduction of *Herpesvirus saimiri* immortalized T cells as a model for gene therapy in primary immunodeficiencies. Gene Ther.

[18] Zsindely N, Pankotai T, Ujfaludi Z (2009). The loss of histone H3 lysine 9 acetylation due to dSAGA-specific dAda2b mutation influences the expression of only a small subset of genes. Nucleic Acid Res.

[19] Carvalho PH, Daibert AP, Monteiro BS (2013). Differentiation of adipose tissue-derived mesenchymal stem cells into cardiomyocytes. Arq Bras Cardiol.

[20] Haberzettl P, Lee J, Duggineni D (2012). Exposure to ambient air fine particulate matter prevents VEGF-induced mobilization of endothelial progenitor cells from the bone marrow. Environ Health Perspect.

[21] Li Y, Chu JS, Kurpinski K (2011). Biophysical regulation of histone acetylation in mesenchymal stem cells. Biophys J.

[22] Passier R, Van Laake LW, Mummery CL (2008). Stem-cell-based therapy and lessons from the heart. Nature.

[23] Li L, Zhu J, Tian J, Liu X, Feng C (2010). A role for Gcn5 in cardiomyocyte differentiation of rat mesenchymal stem cells. Mol Cell Biochem.

[24] Colussi C, Berni R, Rosati J (2010). The histone deacetylase inhibitor suberoylanilide hydroxamic acid reduces cardiac arrhythmias in dystrophic mice. Cardiovasc Res.

[25] Pufahl L, Katryniok C, Schnur N (2012). Trichostatin A induces 5-lipoxygenase promoter activity and mRNA expression via inhibition of histone deacetylase 2 and 3. J Cell Mol Med.

[26] Grant PA, Duggan L, Côté J (1997). Yeast Gcn5 functions in two multisubunit complexes to acetylate nucleosomal histones: characterization of an Ada complex and the SAGA (Spt/Ada) complex. Genes Dev.

[27] Chakraborty A, Paul BD, Nagaraja V (2007). Bacteriophage Mu C protein is a new member of unusual leucine zipper-HTH class of proteins. Protein Eng Des Sel.

[28] Kosaka N, Kodama M, Sasaki H (2006). FGF-4 regulates neural progenitor cell proliferation and neuronal differentiation. FASEB J.

[29] Mishra R, Vijayan K, Colletti EJ (2011). Characterization and functionality of cardiac progenitor cells in congenital heart patients. Circulation.

[30] Moretti A, Bellin M, Jung CB (2010). Mouse and human induced pluripotent stem cells as a source for multipotent Isl1^+^cardiovascular progenitors. FASEB J.

